# Methicillin-Resistant *Macrococcus bohemicus* Encoding a Divergent SCC*mecB* Element

**DOI:** 10.3390/antibiotics9090590

**Published:** 2020-09-10

**Authors:** Geoffrey Foster, Gavin K. Paterson

**Affiliations:** 1 SRUC Veterinary Services, Inverness IV2 5NA, UK; geoffrey.foster@sac.co.uk; 2 Royal Dick School of Veterinary Studies and The Roslin Institute, University of Edinburgh, Edinburgh EH25 9RG, UK

**Keywords:** *Macrococcus*, SCC*mec*, *mec* genes *mecB*, methicillin-resistance, veterinary microbiology, antimicrobial resistance

## Abstract

A methicillin-resistant *Macrococcus* isolate from canine otitis, H889678/16/1, was whole-genome sequenced using HiSeq technology to identify the species, antimicrobial resistance determinates and their genomic context. H889678/16/1 belonged to the newly described species *Macrococcus bohemicus*. It encoded *mecB* within a novel SCC*mec* element most similar to that of *Macrococcus canis* KM45013^T^. This SCC*mec*_H889678/16/1_ element also encoded *blaZ_m_* and *fusC*, but no other resistance determinates were found in the H889678/16/1 genome. The *ccrA* and *ccrB* recombinase genes within SCC*mec*_H889678/16/1_ were distinct from those previously described in staphylococci and macrococci and therefore designated here as *ccrAm3* and *ccrBm3*. Our study represents, to the best of our knowledge, the first description of *mecB* being encoded by *M*. *bohemicus* and of methicillin resistance in this species. Furthermore, the SCC*mec* described here is highly dissimilar to other such elements and encodes novel *ccr* genes. Our report demonstrates a wider distribution of *mecB* among *Macrococcus* species and expands the genomic context in which *mecB* may be found. The potential for dissemination of *mec* genes from *Macrococcus* to related but more pathogenic *Staphylococcus* species highlights the need to understand the epidemiology of these genes in macrococci.

## 1. Introduction

The genus *Macrococcus* is closely related to *Staphylococcus* and consists of eleven species typically found as commensals in a range of animal hosts [1]. However, there is a growing appreciation that some macrococci may also act as opportunistic pathogens in different animals. For instance, *M*. *caseolyticus* has been isolated from mastitis in dairy cattle [2,3], canine dermatitis [4], canine otitis [2], an outbreak high-mortality systemic infection in broiler chickens [5], ovine abscesses [6] and cases of embryo mortality in greater white-fronted geese (*Anser albifrons*) [7]. *Macrococcus canis* has also been isolated from a range of canine infections [4] and a small number of isolates of different *Macrococcus* species have come from human clinical samples, suggesting a potential role, albeit infrequently, in human infections as well [8].

As with staphylococcal species, macrococci can acquire methicillin resistance through *mec* genes which encode an alternative penicillin-binding protein, PBP2a [9]. While PBP2a is encoded by *mecA* in *Staphylococcus*, and to a lesser extent by *mecC* [10], methicillin resistance in *Macrococcus* is encoded by the *mec* gene alleles *mecB* and *mecD*. *mecB* has been reported from *M*. *caseolyticus* [5,11,12,13], *M*. *canis* [4,14] and *Macrococcus goetzii* [8,15]. To date, *mecD* has only been reported from *M*. *caseolyticus* [2,3,4,12].

*mecB* has been found to be encoded by various genetic elements in *Macrococcus;* different SCC*mec* elements [11,13], different plasmids [11,14,16] and different ΨSCC elements [8,14]. Importantly, there has been a single report of a human isolate of *Staphylococcus aureus* encoding *mecB* [17] on a plasmid almost identical to a *M*. *canis* plasmid also encoding *mecB* [14]. This raises the strong possibility of the exchange of methicillin resistance determinates between these two genera and highlights the need to better understand the epidemiology and genomics of *mec* genes among *Macrococcus*.

*Macrococcus bohemicus* was first described in 2018 on the basis of a single isolate cultured from a human traumatic knee wound sample collected in 2003 in the Czech Republic [8]. Subsequently, a second *M*. *bohemicus* isolate coming from bovine milk in the Republic of Ireland and collected in 2017 has been described [18]. Both isolates have been genome sequenced.

To the best of our knowledge, no *mec* gene or methicillin resistance has been described in *M*. *bohemicus*, and herein we describe the first example of such, a canine otitis isolate H889678/16/1 encoding *mecB* within a distant SCC*mec* element and carrying novel *ccrA* and *ccrB* alleles, designated here as *ccrAm3* and *ccrBm3*.

## 2. Results and Discussion

### 2.1. Isolation and Whole-Genome Sequencing of Methicillin-Resistant M. bohemicus H889678/16/1

H889678/16/1 was isolated from mixed growth cultured from a canine otitis sample collected from a cocker spaniel in Scotland in 2016. Also isolated were an *Enterobacter* sp., yeast (likely *Malassezia pachydermatis*) and *Aerococcus viridans*. H889678/16/1 was identified phenotypically as a presumed *Macrococcus* sp. and considered to be methicillin resistant on the basis of resistance to oxacillin when tested by Vitek2. H889678/16/1 was also resistant to benzylpenicillin and fusidic acid but susceptible to all the other antimicrobials tested. H889678/16/1 was whole-genome sequenced using HiSeq technology to resolve its identity to the species level and to determine the genetic basis for methicillin resistance. The resultant assembled draft genome consisted of 49 contigs totalling 2,497,285 bp in length, with a GC% content of 33.89%. The average genome coverage was 42.9-fold. H889678/16/1 was identified as belonging to *M*. *bohemicus* using the Type Strain Genome Server [19] and showed a dDDH value of 83.3 against *M*. *bohemicus* type strain CCM 7100.

### 2.2. M. bohemicus H889678/16/1 Encodes mecB within a Novel SCCmec Element

**Figure 1:**
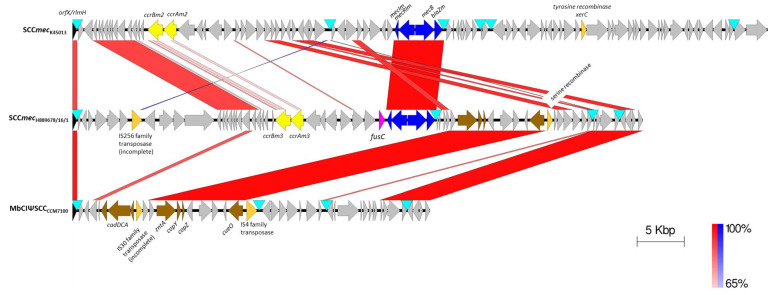
Schematic comparison of SCC*mec* and ΨSCC elements in *M*. *canis* KM45013^T^ (**top**), *M*. *bohemicus* H889678/16/1 (centre) and *M*. *bohemicus* CCM7100^T^ (**below**). The sequences used are as follows: *M*. *canis* KM45013^T^ CP021059.1 region: 31942 … 105740; *M*. *bohemicus* H889678/16/1 JACEGF000000003.1 region: 81206 … 141735; *M*. *bohemicus* CCM7100^T^ PZJG01000007.1 region: 14792 … 52744. Regions of homology are represented by bands connecting the genomes sequences, with the percentage identity key shown on the key. Red denotes normal sequence alignment (N); blue denotes inverted sequence alignment (I). Selected features are coloured and labelled. Direct repeats are indicated by blue triangles. Colouring of genes denotes the following: *orfX*/*rmlH*, black; *mec* gene complex, blue; *ccr* genes, yellow; mobile elements, gold; genes putatively involved in heavy metal resistance, brown.

ResFinder analysis of the H889678/16/1 genome showed that it encoded *mecB* and *fusC*. Further analysis showed that *mecB* was within a *mec* gene complex with *blaZm,* but no other antimicrobial resistance determinates were apparent in the genome. All three genes, *mecB*, *blaZ_m_* and *fusc*, were located on a single contig 265 kbp in size (JACEGF000000003) and encoded within a SCC*mec* element in the *orfX*/*rlmH* region. The insertion of the element into the *orfX*/*rlmH* region has to date been a reliable indication of a chromosomal location for SCC*mec* elements. Additional evidence for this being the case in H889678/16/1 are the large size of the SCC*mec*-containing contig, the absence of any plasmid features in this contig as detected by PlasmidFinder and the presence of numerous housekeeping genes on this contig, including those likely to be essential for viability, such as *gyrA* and *gyrB*. This SCC*mec* element, designated as SCC*mec*_H889678/16/1_, was most similar to, but distinct from, the *mecB*-encoding SCC*mec* of *M*. *canis* KM45013^T^ (Figure 1) [13]. SCC*mec*_H889678/16/1_ is also distinct from the ΨSCC element found in the *M*. *bohemicus* type strain CCM7100 that lacks a *mec* gene complex and *ccr* genes (Figure 1) [8]. The only other described *M*. *bohemicus* isolate, DPC 7215 [18], also lacks any *mec* gene and possesses an *orfX*/*rlmH* region distinct from that of H889678/16/1 (data not shown). SCC*mec*_H889678/16/1_ is 57,612 bp in size, as defined by the length from the two outermost direct repeats inclusive (Figure 1). The *mec* gene complex of *mecI*, *mecR1*, *mecB* and *blaZ_m_* in SCC*mec*_H889678/16/1_ was highly conserved with those of SCC*mec*_KM45013_, with each gene pair sharing 98–99% nucleotide identity. The other large region conserved between these two elements is a series of eight genes located near the *ccr* genes which encode the DNA repair protein RadC, a helix-turn-helix domain protein and six hypothetical proteins. This region of similarity extends into portions of the two flanking genes and, in the apparent absence of adjacent mobile genetic elements, may indicate their acquisition by homologous recombination. SCC*mec*_H889678/16/1_ also contained blocks of similarity with the ΨSCC element found in the *M*. *bohemicus* CCM7100^T^, indicating a mosaic structure likely arising through multiple horizontal genetic transfer events.

### 2.3. SCCmec_H889678/16/1_ Encodes Novel Recombinase Genes ccrA3m and ccrB3m

**Figure 2:**
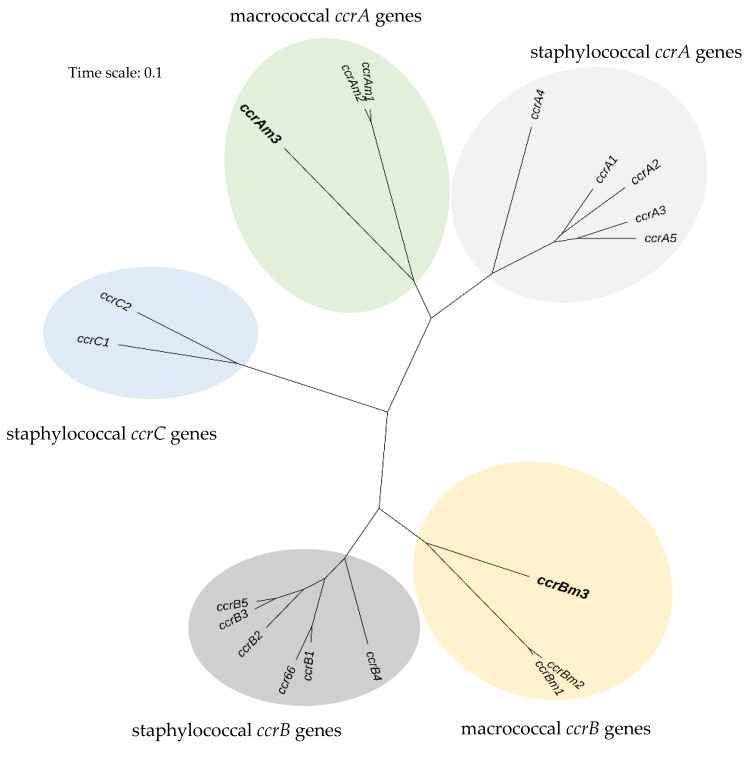
Phylogenetic relationships among *ccr* genes in *Macrococcus* and *Staphylococcus*. *ccrAm3* and *ccrBm3* from this study are highlighted in bold. Nucleotide accessions used are as follows: *ccrAm1* and *ccrBm1 M*. *caseolyticus* JCSC7096, GenBank accession no. AB498756; *ccrAm2* and *ccrBm2 M*. *caseolyticus* KM45013, GenBank accession no. HG970732; *ccrA1* and *ccrB1 S*. *aureus* NCTC10442, GenBank accession no. AB033763; *ccrA2* and *ccrB2 S*. *aureus* N315, GenBank accession no. BA000018; *ccrA3* and *ccrB3 S*. *aureus* 85/2082, GenBank accession no. AB037671; *ccrA4* and *ccrB4 S*. *aureus* HDE288, GenBank accession no. AF411935; *ccrA5* and *ccrB5 S*. *pseudintermedius* KM241, GenBank accession no. AM904731; *ccrB6 S*. *aureus* JCSC6945, GenBank accession no. AB505630; *ccrC1 S*. *aureus* JCSC6082, GenBank accession no. AB373032; *ccrC2 S*. *aureus* strain BA01611 GenBank accession no. KR187111. There were a total of 1804 positions in the final dataset. Scale bar indicates number of substitutions per site.

A notable feature of the SCC*mec*_H889678/16/1_ is the relative lack of similarity shown by the *ccr* genes with those of SCC*mec*_KM45013_ (Figure 1), with the *ccr* genes being responsible for the excision and integration of SCC*mec* elements in and out of the genome. Indeed, SCC*mec*_H889678/16/1_ *ccr* genes share limited nucleotide identity with known *Macrococcus* and *Staphylococcus ccr* genes, 43.5–63.0% in the case of *ccrB* and 41.5–54.4% in the case of *ccrA*. Following precedent [11,13], we propose the designation of the SCC*mec*_H889678/16/1_ *ccr* genes as *ccrAm3* and *ccrBm3*. A phylogenetic analysis of *ccr* genes highlights the distinctness of *ccrAm3* and *ccrBm3* (Figure 2). Whilst *ccrAm3* and *ccrBm3* belong to their respective *Macrococcus ccr* gene branches, they are distant to these counterparts, with *ccrAm3* and *ccrBm3* closest to the ancestral forms of the macrococcal *ccr* genes.

In conclusion, we report the first example, to the best of our knowledge, of methicillin resistance and *mecB* in the newly described species *M*. *bohemicus.* This is only the third description of a *M*. *bohemicus* isolate and appears to be the first isolation from a dog. The *mecB* gene in this isolate is encoded within a distinct SCC*mec* element containing novel *ccr* alleles. This expands our knowledge on the distribution and genomic context of methicillin resistance determinates among *Macrococcus* which themselves are opportunistic pathogens, but which may also act as a genetic reservoir for the more pathogenic and related *Staphylococcus*.

## 3. Materials and Methods

### 3.1. Antimicrobial Sensitivity Testing

Antimicrobial sensitivity testing was performed using Vitek2 (bioMérieux, Basingstoke, UK) following the manufacturer’s instructions. Using the Vitek2 AST-P634 card, the antimicrobials tested were as follows: cefoxitin (screen), benzylpenicillin, oxacillin, gentamicin, ciprofloxacin, inducible clindamycin resistance, erythromycin, clindamycin, linezolid, teicoplanin, vancomycin, tetracycline, nitrofurantoin, fusidic acid, mupirocin, chloramphenicol, rifampicin and trimethoprim with interpretation performed using The Clinical and Laboratory Standards Institute criterion (2015) for coagulase-negative staphylococci.

### 3.2. Whole-Genome Sequencing

Whole-genome sequencing was performed by Microbes NG (University of Birmingham, Birmingham UK) using Illumina technology with 2 × 250 bp paired-end reads. Genomic DNA was purified with solid-phase reversible immobilization beads, and libraries were prepared using Nextera XT Library Prep Kit (Illumina, San Diego, CA, USA) following the manufacturer’s protocol with the following modifications: two nanograms of DNA instead of one were used as input, and PCR elongation time was increased to 1 min from 30 seconds. Reads were trimmed using Trimmomatic version 0.30 [20], using a sliding window quality cut-off of 15. Genome assembly was done *de novo* using SPAdes, version 3.7, with default parameters for 250 bp Illumina reads [21] and annotated by the NCBI Prokaryotic Genome Annotation Pipeline [22].

### 3.3. Genome Analysis

Genome-based identification was done using the Type Strain Genome Server [17] (https://tygs.dsmz.de/). Acquired resistance genes were identified using ResFinder-3.1 [23] employing the thresholds of 60% for percentage identity and minimum length of 60%. Visual inspection and formatting of the genome for Figure 1 was performed using Artemis 17.0.1 [24]. Schematic comparison of *mecB* regions was performed using EasyFig 2.2.5 [25]. Plasmid features were searched for using PlasmidFinder 2.0 [26] with the thresholds of 60% for percentage identity and minimum length of 60%. The phylogenetic relationship of *ccr* genes was assessed in MEGA X [27] using a single representative of each type (nucleotide accession numbers provided in Figure 2), aligning the nucleotide sequences with MUSCLE and producing a maximum-likelihood tree using a general time-reversible (GTR) model. The final tree was produced using the Interactive Tree of Life (iTOL) [28].

## 4. Nucleotide Accession Numbers

The whole-genome sequencing reads and annotated assembly of *M*. *bohemicus* H889678/16/1 are available under the GenBank accession numbers SRR12266687 and JACEGF000000000, respectively.
